# Energy as a Common Currency for Understanding Embodied Evolution

**DOI:** 10.34133/cbsystems.0680

**Published:** 2026-09-16

**Authors:** Yue Xie, Yi Zhang, Tao Sun, Fumiya Iida

**Affiliations:** ^1^ Department of Computer Science, Loughborough University, Loughborough LE11 3TT, UK.; ^2^ Key Laboratory of Mechanism Theory and Equipment Design of Ministry of Education, Institute for Embodied Intelligence, Tianjin University, Tianjin 300354, China.; ^3^ Institute for Embodied Intelligence, Tianjin University, Tianjin 300350, China.; ^4^ Bio-Inspired Robotics Lab, Department of Engineering, University of Cambridge, Cambridge CB2 1TN, UK.; ^5^ Bio-Inspired Robotics Lab, Department of Precision Engineering, Graduate School of Engineering, The University of Tokyo, Tokyo 133-0033, Japan.

## Abstract

Unlike living organisms, which persist by balancing energy intake, expenditure, and maintenance, embodied robots are often evaluated primarily by task performance, even when morphology, control, and environment jointly determine what can be sustained over time. This task-centered evaluation supports engineering benchmarks but can obscure embodied costs, limit comparability across heterogeneous embodiments, and weaken biological interpretation when robots and simulations are used as model systems. This Review synthesizes biological energetics and biomechanics to argue that open-system energy accounting provides a common currency for embodied evolution. Under this framing, adaptive success (fitness) is interpreted as persistence under energetic constraint, enabling commensurate comparison of regulation, structural adaptation, and structural plasticity across environments with different energetic structure. A minimal accounting framework and an illustrative experimental paradigm are presented to show how shared accounting yields interpretable differences between adaptive responses beyond task semantics. This paper concludes by discussing implications for interpreting morphological and control complexity and by outlining open challenges and future directions for energy-aware embodied evolution using robots as controlled platforms for biology-facing hypothesis testing.

## Introduction

Embodied evolution concerns how adaptive behavior emerges in systems whose capabilities are inseparable from physical form and environmental embedding. Across bio-inspired robotics, artificial life, biomechanics, and embodied cognition, a recurring lesson is that intelligence is not localized solely in a controller but arises from the coupled dynamics of brain, body, and environment [1–4]. Evolutionary robotics has demonstrated this coupling by showing that morphology and control can co-evolve to produce effective behavior [5–10]. At the same time, an increasingly important reverse direction has emerged: robotic and simulation platforms can serve as experimental tools for probing biological form: function relationships and evolutionary hypotheses, particularly when direct biological evidence is incomplete or difficult to manipulate [11,12]. Recent bio-inspired robotics synthesis further emphasizes that energy-saving strategies in animals and plants are shaped by environment-specific constraints and uncertainty, and that extracting these principles can guide robot design for extended operativity under resource limitation [13].

Despite this cross-disciplinary development, a central methodological question remains unresolved: what should count as adaptive success (fitness in evolutionary computation) in embodied evolution? Much of the literature still defines success through task-centered objectives such as distance travelled, goal-reaching, reward accumulation, or trial completion [8–10,14]. Such objectives are valuable for engineering benchmarks, but they are often insufficient for scientific comparison when embodiments differ, environments change, or the goal is to understand adaptation as sustained organization rather than episodic achievement. Here, sustained organization means the continued operation of a body–controller–environment system under persistent energetic cost and constraint, whereas episodic achievement means success on a bounded task instance, such as reaching a target or completing a trial. Thus, the evaluation metric is not a neutral reporting choice: it determines whether adaptive success is interpreted primarily as task achievement, persistence under constraint, or both.

In this paper, we argue that energy should serve as a common currency for understanding embodied evolution. We use energy as the common currency and viability as the interpretive criterion: energetic viability denotes the capacity of an embodied agent to sustain interaction over time while maintaining a positive energy reserve under the ongoing costs of acting, maintaining its body, and interacting with the environment. Energy accounting does not fully decouple control, morphology, and environment as independent causes. Instead, it provides a shared physical bookkeeping interface through which their energetic consequences can be made explicit: controller contributions appear through action and actuation costs, body contributions through morphology-dependent expenditure or maintenance terms, and environmental contributions through intake opportunities, dissipation, and resource accessibility. This stance is grounded in the physical view of embodied agents as open systems that exchange energy with their environment, and it aligns with biological intuition: organisms persist under resource limitation by balancing intake, expenditure, reserve dynamics, allocation, and maintenance ([15], Chapters 1 and 2, and [16], Chapters 1 and 2). It also aligns with morphological computation perspectives, which emphasize that morphology and physical dynamics can reduce controller burden and reshape what must be explicitly controlled [17,18]. Using explicit energy accounting, evaluation can therefore report both task achievement and persistence under energetic constraint rather than reducing adaptive success to task score alone.

Explicit energy accounting can strengthen the role of robots and simulations as biological models by making acting and persistence costs comparable across embodiments. Webb [12] emphasizes that the value of robotic models depends on stating what is represented, what is abstracted, and the scope of claims. We build on this perspective by proposing that energetic accounting constitutes an additional and often missing modeling dimension: whether a model makes the costs of acting, persisting, and adapting commensurable across embodiments. This is particularly important in embodied evolution, where key variables operate on different physical scales and require careful normalization. Existing dimensionless metrics, such as cost of transport in locomotion or Strouhal-number-based measures in swimming and flying, provide valuable normalized comparisons for specific physical regimes. However, these metrics usually target particular performance or efficiency relations. Energy accounting is proposed here as a complementary open-system bookkeeping layer that tracks intake, expenditure, overhead, and persistence across adaptive regimes, rather than as a replacement for established domain-specific metrics.

**Fig. 1.**
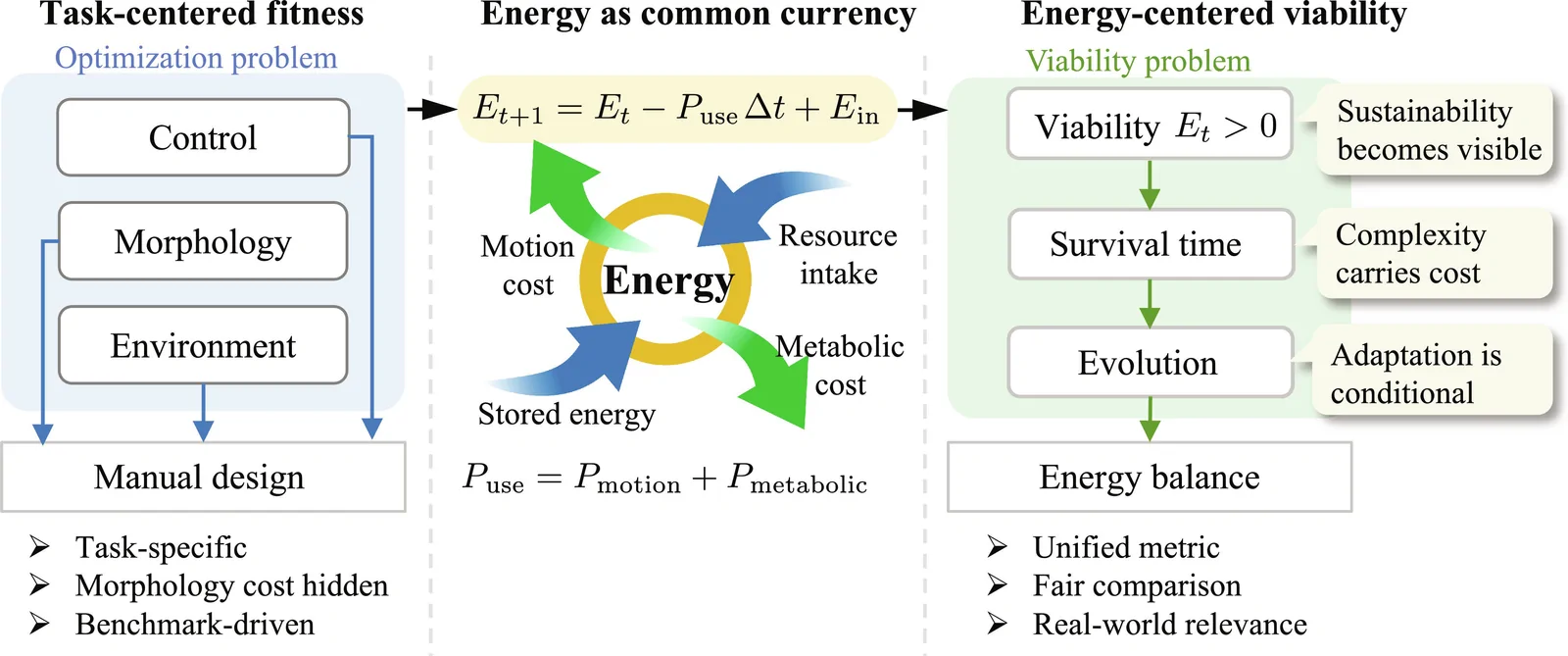
Shift from task reward to energy-centered viability. Survival time under an energy budget compares adaptation regimes on a common currency.

Fig. 1 provides a supporting illustration of the paper's central claim. It contrasts task-centered scoring with energy-centered persistence under constraint and shows how explicit energy accounting enables regulation, structural adaptation, and structural plasticity to be compared under a shared interpretive framework [6,11,19].

For clarity, key terms are defined as follows. Evolution refers to population-level change in heritable traits across generations under selection. Adaptation denotes changes that improve persistence or performance under a specified set of constraints; in this review, it can occur through controller change, morphology change, or both, depending on the regime. Energy refers to an explicitly tracked physical quantity in an open system, represented through intake and expenditure in the accounting model. Task-centered evaluation scores agents primarily by externally specified task achievement (e.g., targets reached), whereas persistence under energetic constraint interprets success by how long an embodied system continues operating under explicit accounting of expenditure, overhead, and morphology-dependent costs. An organizing variable is a quantity that provides a unifying explanatory axis across heterogeneous mechanisms; here, it means that energetic constraint systematically structures feasible behavior and embodiment. Homeostasis refers to maintaining key variables within viable bounds despite disturbance, and allostasis refers to maintaining stability through change with cumulative cost (allostatic load). Structural plasticity denotes within-lifetime changes to embodiment (e.g., remodeling/repair), contrasted with structural adaptation across design iterations or generations. These definitions are used consistently throughout the manuscript.

## Robots and Simulations as Biological Models and Defining Adaptive Success

Robots and simulations are increasingly used as model systems for investigating biological behavior, morphology–function relationships, and evolutionary hypotheses [3,4,11]. Here, a model system means a controlled robotic or computational system used to represent selected aspects of a biological target, rather than a full replica of the organism. Its scientific value depends on clarity about what is represented, what is abstracted away, and how conclusions should generalize. A useful methodological lens is provided by Webb [12], who argues that robots can be good models of biological behavior if they are treated as models rather than replicas and if their relationship to the biological target is specified along explicit dimensions. Under this interpretation, disagreement about whether a robot is a good biological model often arises because different readers assume different biological targets, levels of abstraction, or intended claims.

This review aligns with views that treat models as representational instruments tailored to particular explanatory aims, involving trade-offs among realism, generality, and precision [20,21]. It also resonates with computational science methodology emphasizing that credible simulation-based inference requires transparent assumptions and appropriate verification and validation practices [22,23]. These issues are especially critical for embodied evolution because the model–target mismatch is distributed across multiple coupled subsystems. Here, mismatch refers to discrepancies between the robotic or simulated system and the biological target system in the variables that mediate embodied interaction. These include body–environment variables such as contact geometry, friction, compliance, and substrate properties; sensorimotor variables such as sensing bandwidth, noise, actuation limits, latency, and control update rate; and organism-level variables such as energetic expenditure, maintenance burden, resource intake, and failure or repair dynamics. Such mismatches matter because embodied evolutionary outcomes depend not only on controller performance, but also on how body, control, and environment jointly determine persistence over time ([16], Chapters 1 and 2, and [15], Chapters 1 and 2).

This criticality is specific to embodied evolution because the evaluated phenotype is not a controller alone, but a coupled body–controller–environment organization. Empirical work in evolutionary robotics has repeatedly shown that performance and transfer depend on this coupling: evolved controllers can fail when simulated dynamics differ from real hardware, morphology–control co-optimization can be sensitive to hardware limitations such as reduced actuator capability, and evolved morphology–controller configurations can vary important across physical environments [24,25]. Thus, mismatch is not merely a reporting inconvenience; it can alter the adaptive mechanism selected by evolution, the apparent robustness of a solution, and the biological interpretation assigned to the model. For this reason, embodied evolution requires explicit reporting of which variables are modeled, which are abstracted, and which costs are included in evaluation.

Explicit energy accounting strengthens robots and simulations as biological models not by making them automatically more realistic, but by making their claims more interpretable and comparable. Many biology-facing questions concern persistence under energetic constraint: how maintenance burdens, action costs, and environmental structure shape feasible behavior and embodiment. If a robotic model reports only task score, it can be unclear whether observed differences arise from morphology, control, or unaccounted embodied costs. By contrast, tracking intake, expenditure, and persistence costs makes these constraints explicit and commensurate, allowing hypotheses about allocation trade-offs and condition-dependent adaptive mechanisms to be tested under controlled changes to embodiment and environment. This extends Webb's modeling perspective by clarifying which biological aspect is being represented here: energetic constraint as an organizing condition for sustained adaptation.

Within this modeling perspective, embodied evolution raises a concrete question: what should count as adaptive success when body, control, and environment jointly shape behavior over time? In evolutionary robotics and artificial life, success is most commonly operationalized through task-centered objectives such as locomotion distance, target reaching, or reward accumulation [8–10]. This tradition has generated influential demonstrations of evolved behavior and co-adaptation. Sims' virtual creatures showed that simulated evolution can produce coupled morphology–control strategies for locomotion and competition in physically simulated worlds [5]. Lipson and Pollack [26] demonstrated that evolved robot morphologies can be automatically designed and physically fabricated, linking evolutionary design to real robotic embodiment. Later soft-robot evolution studies, including voxel-based soft robots, showed that morphology and control can be co-optimized in deformable bodies and task environments [7]. These studies were foundational, but their evaluation was primarily organized around task achievement, such as locomotion performance or task success. As a result, they provide limited information about long-horizon energetic persistence, maintenance burden, resource intake, or whether similar task outcomes arise from energetically different adaptive mechanisms. At the same time, when robots and simulations are used as biology-facing model systems, task performance can be an incomplete proxy for long-horizon persistence, robustness, and sustained adaptivity, because sustained embodied organization depends on physical constraint, reserve dynamics, allocation, and maintenance rather than episodic achievement alone ([16], Chapters 1 and 2, and [15], Chapters 1 and 2) [11].

Throughout this review, viability denotes the capacity of an embodied system to remain functional over time under persistent cost and constraint. This is the conceptual definition used throughout the review. When a formal operationalization is required, we specify the corresponding viability criterion explicitly in the “Energy as a Common Currency for Embodied Evolution” section and in the illustrative paradigm in the “Illustrative Experimental Paradigm and Evidence” section.

Task objectives are indispensable for engineering benchmarks; the concern raised here is construct validity and fairness when task score is used as the primary proxy for sustained embodied adaptation across heterogeneous embodiments. Task performance can correlate with energy use in some settings, but it does not generally subsume baseline overhead, morphology-dependent persistence costs, or the energetic consequences of different embodiment choices. To make this point concrete rather than rhetorical, the illustrative paradigm in the “Illustrative Experimental Paradigm and Evidence” section reports both a task metric (targets reached) and persistence under energetic constraint (survival time) under shared accounting, showing how regimes can align in task achievement yet diverge in persistence once embodied costs are made explicit.

Several simple scenarios illustrate this limitation. In locomotion, 2 agents may travel the same distance, but one may rely on high-frequency active correction while the other exploits passive body dynamics; the task score is identical, but the energetic burden and robustness implications differ. In manipulation or target reaching, 2 arms may reach the same number of targets, but a heavier or less efficient morphology may deplete its energy reserve faster under the same intake rule. In changing environments, a controller that maximizes short-term reward on a fixed terrain may fail when friction, load, or resource accessibility changes, whereas a morphology or policy with lower peak reward may persist longer because it reduces continual compensation. These cases show that performance-based evaluation remains useful for measuring task achievement, but it does not by itself distinguish episodic success from sustained embodied organization.

These scenarios can be summarized as a construct-validity condition rather than as a rejection of task benchmarking. A task score is an incomplete basis for embodied evolution when the intended construct is not episodic achievement but sustained adaptive organization. This incompleteness arises when 3 conditions hold: the agent must persist over time under continuing cost; different body–controller–environment configurations can produce the same task score through different mechanisms, and the scoring rule does not account for the energetic liabilities that determine whether the organization can continue operating. Under these conditions, task performance remains useful, but it is not sufficient to distinguish a strategy that merely achieves the task from one that remains viable under embodied constraint. This is the logical basis for introducing explicit energy accounting as a complementary evaluation dimension.

More broadly, task objectives are less suited to embodied evolution when the phenomena of interest include sustainability, failure recovery, trade-off structure, and adaptive mechanism selection. First, task rewards can be sparse, delayed, or deceptive in high-dimensional embodied search spaces, leading to brittle strategies that exploit benchmark idiosyncrasies rather than robust organization [27–29]. Second, task fitness often fails to make embodied liabilities explicit, including energetic expenditure, maintenance burden, reserve allocation, and the cost of continual compensation by control ([15], Chapter 2) [30,31]. Third, task-centric evaluation makes it difficult to compare heterogeneous contributions: when 2 agents achieve similar task scores, it can be unclear whether sustained function arises primarily from controller sophistication, morphological advantage, or exploitation of environment structure ([32], Chapters 1 to 3, 8, and 13) [1,2].

These limitations are reflected in methodological shifts that relax strict objective maximization. Novelty search abandons task objectives to avoid deception and stimulate discovery [27], and resilience-focused robotics emphasizes that peak benchmark performance is insufficient if the agent cannot remain functional under perturbation, damage, or regime change [6,19]. The shared insight is that evaluation should help explain how solutions remain viable and under what conditions, not merely whether they succeed once under a fixed scoring rule.

Embodied evolution introduces an additional modeling dimension that is often left implicit but is crucial for cross-embodiment comparison: explicit energetic accounting of expenditure, intake, and persistence costs. The framework and notation used in this review are defined formally in the “Energy as a Common Currency for Embodied Evolution” section.

Making energetic accounting explicit also improves comparability and reporting. If energy is treated as a modeling dimension, studies should state what is accounted for, how intake is represented, and how viability is computed, so conclusions can be interpreted and compared across embodiments and platforms [22].

Table 1 summarizes these modeling dimensions and shows how they can be used as a reporting checklist for embodied evolution. The table is not intended to rank models by realism; rather, it clarifies what a model represents, which subsystems are included, what claims are meant to generalize, and whether energetic intake and expenditure are part of the modeled phenomenon. For example, in the planar-arm paradigm used later in the “Illustrative Experimental Paradigm and Evidence” section, the model deliberately abstracts biological tissue and detailed metabolism, but specifies the body, controller, reaching task, target-based intake rule, action-dependent expenditure, and maintenance-like cost. Under the table's dimensions, the scenario therefore supports claims about persistence under energetic constraint and regime comparison, but not claims about organism-specific physiology. This illustrates how the dimensions function in practice and how energy accounting clarifies the scope of biological interpretation.

**Table 1.** Modeling dimensions for using robots and simulations as biological model systems, extended with energy and viability accounting for embodied evolution

Modeling dimension	Role in embodied evolution
Representational fidelity	Which physical and biological details are represented, such as material properties, sensing, actuation, contact, and environmental structure.
Subsystem coverage	Which interacting subsystems are included, such as body, controller, sensors, development, energetic intake, and environment coupling.
Scope of generalization	Whether the model supports generic principles, organism-specific claims, task-specific claims, or regime-specific claims.
Explanatory target	Whether the model is used to predict outcomes, explain mechanisms, or test hypotheses about body–control–environment organization.
Measurement mode	Whether conclusions rely on quantitative outputs, qualitative behavioral patterns, mechanistic traces, or combinations of these.
Biological grounding of assumptions	Whether modeling assumptions are biologically motivated, engineering conveniences, or explicit abstractions made for controllability.
Validity domain	The conditions under which conclusions should hold, including bodies, environments, tasks, energetic regimes, and perturbation ranges.
Energy and viability accounting	Whether intake, expenditure, action costs, overhead, maintenance-like costs, and adaptation costs are explicitly represented so that persistence can be compared across embodiments and environments.

**Fig. 2.**
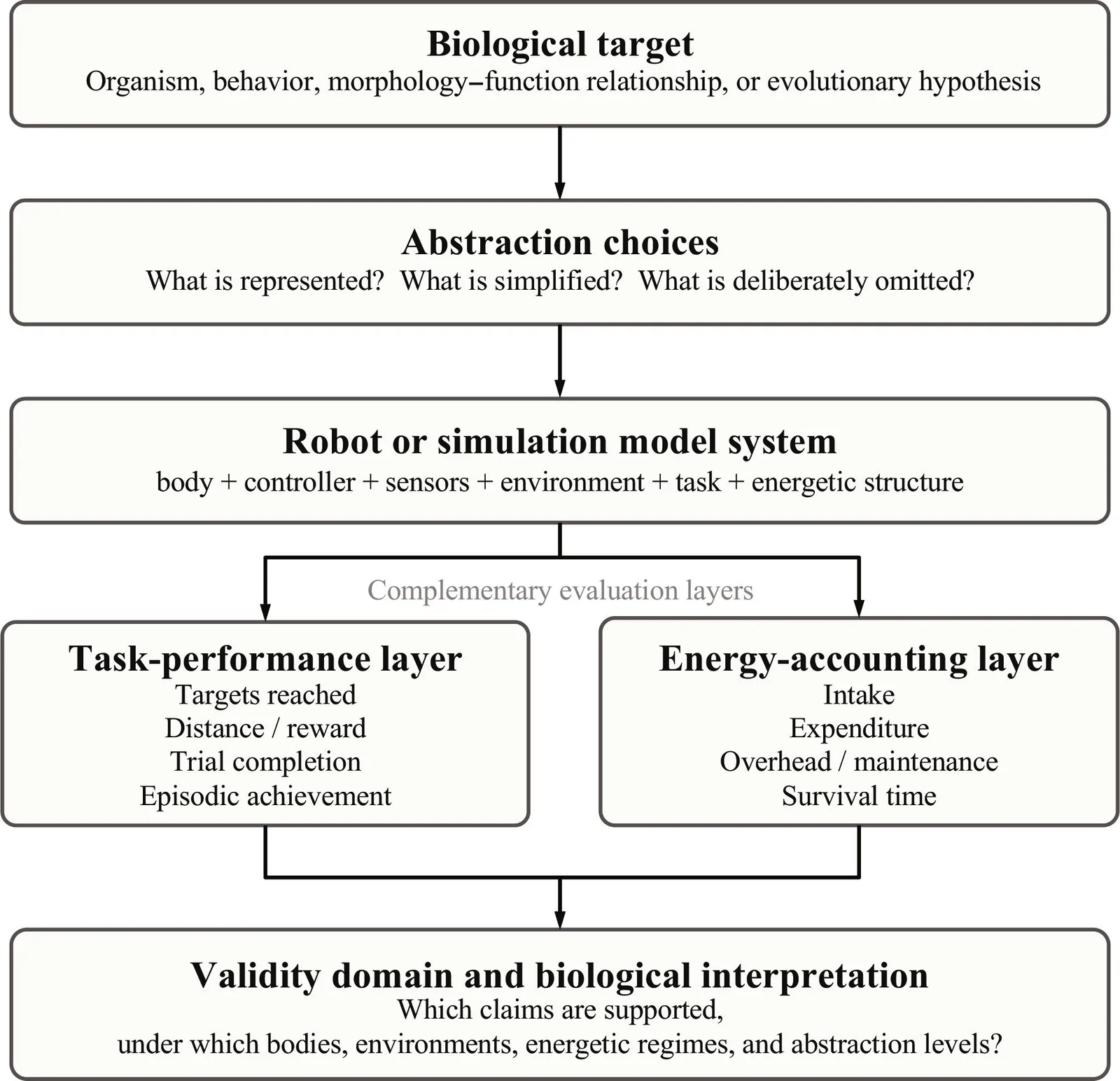
Schematic use of the modeling dimensions in Table 1. A biological target is represented through explicit abstraction choices, evaluated through complementary task-performance and energy-accounting layers, and interpreted within a stated validity domain.

Fig. 2 schematizes how the modeling dimensions in Table 1 are used. A biological target is not copied directly into a robotic or simulation model; rather, selected aspects are represented through explicit abstraction choices. Task performance reports episodic achievement, whereas energy and viability accounting reports persistence under cost. The validity domain then constrains which biological interpretations can be supported. The figure therefore complements Table 1 by showing how representational fidelity, subsystem coverage, explanatory target, measurement mode, and energy accounting jointly determine the scope of biological claims.

## Energy in Biology as the Organizing Variable

Because energy is a physical quantity, its role as a common currency for embodied evolution is grounded first in thermodynamics and open-system reasoning. Its biological relevance arises because living systems instantiate these physical constraints through metabolism, resource intake, allocation, maintenance, and adaptation. Across physiology, ecology, biomechanics, and life-history theory, organisms persist and evolve under continual trade-offs in energy acquisition, conversion, allocation, and maintenance ([33], Chapters 1 and 2) [34] ([15], Chapters 1 and 2, and [16], Chapters 1 and 2). Recent bio-inspired robotics synthesis reinforces the same premise from a design-facing angle: energy-saving strategies in animals and plants are shaped by environment-specific constraints and uncertainty, and these principles can guide robot design for extended operativity under resource limitation [13]. This section distills why energetic constraint organizes persistence and adaptation in living systems, and therefore motivates energy-aware evaluation in robot and simulation model systems.

A defining feature of living systems is that they must continually pay energy to maintain structure and function. Classical physiology framed this as homeostasis, keeping key variables within viable bounds despite external fluctuation ([33], Chapters 1 and 2). More recent accounts emphasize allostasis and allostatic load: adaptation is stability through change that carries cumulative energetic and physiological costs [34]. These concepts matter for embodied evolution models because short-horizon performance can coexist with hidden energetic liabilities that undermine long-horizon viability.

Regeneration and tissue repair provide a clear illustration that adaptation is not free. Repair requires energy and biosynthetic substrates and competes with growth, locomotor performance, and reproduction, producing allocation trade-offs that depend on resource availability. Empirically, echinoderm arm regeneration carries measurable energetic costs [35], and vertebrate tail regeneration can constrain growth under low food availability [36]. At the mechanistic level, regeneration and wound healing are tightly coupled to metabolic state and metabolic reprogramming [37]. For embodied evolution, the methodological lesson is direct: if models aim to capture sustained biological adaptation, repair and remodeling should be treated as explicit energetic commitments rather than free operations.

Dynamic energy budget theory formalizes these principles by treating maintenance as fundamental and by modeling how organisms allocate energy among competing demands such as maintenance, growth, and reproduction ([15], Chapter 2) [38,39]. Closely related energy-allocation perspectives are embedded in life-history theory, where trade-offs arise because energy invested in one function cannot be invested in another [40,41]. The implication for embodied evolution is straightforward: if a model aims to illuminate sustained adaptation rather than episodic task attainment, it must account for persistence costs and represent adaptation as an allocation problem over time, not only an action-execution problem.

Metabolic scaling work motivates the view that energetic throughput constrains feasible morphologies and life histories [31,42,43]. This tradition has produced influential mechanistic accounts and ecological interpretations [44–46], but it is also actively debated. Curvature, taxon dependence, and context dependence challenge the notion of a single universal exponent and emphasize the importance of measurement and statistical treatment [47–51]. For this paper, the lesson is not to impose one correct scaling law on robots; it is that maintenance demands are persistent and body-dependent, and biologically meaningful modeling requires making such energetic liabilities explicit rather than hiding them behind task scores.

Comparative biomechanics makes especially clear why energy is central for understanding embodied evolution. Organisms are embedded in fluids and solids, and physical laws shape feasible motion and structural support. Vogel's synthesis emphasizes that mechanical constraints, material properties, and energetic costs jointly shape viable form and function, thereby connecting evolution to the physics of the environment rather than to task achievement alone ([16], Chapters 1 and 2). This perspective connects naturally to energy-focused bio-inspired robotics: energy-saving strategies are environment-conditioned solutions that exploit body–environment coupling rather than relying solely on energetically costly active compensation [13]. This does not imply that robust or feedback control ignores system–environment coupling; rather, the distinction is where the burden of adaptation is represented. In regulation-dominant strategies, environmental variation is handled primarily through sensing, feedback, and actuation, whereas in morphology- or structure-dominant strategies, part of that burden is shifted into passive dynamics, compliance, geometry, or body–environment coupling. This motivates treating energetic constraint as an organizing variable when comparing embodied strategies across environments, because morphology, behavior, and environment all influence what motions can be sustained and at what ongoing cost.

Locomotion provides a concrete example of how energetic cost shapes behavior and morphology. Classic work established that metabolic cost varies systematically with speed, body size, and gait, and that gait transitions can be understood partly through energetic considerations [52–54]. Comparative and theoretical studies connect limb geometry, posture, elastic storage, and musculoskeletal design to energetic cost and endurance [55,56] ([57], Chapters 1 to 3 and 7), and broader syntheses highlight that locomotor solutions are best understood through the interaction of dynamics, control, and energetic constraints [58,59]. These insights extend naturally to robotics: passive-dynamic and morphology-aware design shows that the physical body can reduce control burden and energetic demand [3,17,60], and mechanistic muscle-energetics models underline that energy consumption is a structured consequence of actuation and force generation [61].

Taken together, these strands motivate treating persistence under energetic constraint as a biologically meaningful lens for interpreting embodied adaptation. This provides the biological rationale for the explicit accounting framework introduced next.

Finally, these biological considerations emphasize that environment structure matters because it shapes both energetic pressure and energetic opportunity through resource accessibility and dissipation [62,63]. This provides the biological motivation for the regime schematic used later in the paper: regulation, structural adaptation, and structural plasticity can be interpreted as distinct responses to energetic and environmental conditions, with repair and remodeling treated as explicit energetic commitments rather than free operations [33,34,37].

## Energy as a Common Currency for Embodied Evolution

Building on the biological grounding, we now define energy as a common currency for embodied evolution. The goal is not to claim a single correct or universal energetic model across organisms and robots, but to specify minimal accounting commitments required for commensurate comparison across embodiments: energy must be explicitly tracked, and both action and persistence must be quantified ([15], Chapters 1 and 2) [30,31]. Because embodied agents operate through fluids, solids, surfaces, and structures, environment structure must also be able to influence viability through intake and/or dissipation ([16], Chapters 1 to 3, 6 to 8, and 23 to 25).

At the most abstract level, persistence under energetic constraint can be operationalized through explicit open-system bookkeeping. An embodied agent carries an internal energy reserve that decreases with use and may increase with intake. A discrete-time update captures this:

$$E\left(t+\Delta t\right)\;=\;E\left(t\right)\;-\;P_{\mathrm{use}}\left(t\right)\Delta t\;+\;E_{\text{in}}\left(t\right)$$ (1)

where $E\left(t\right)$ is the internal energy reserve (J), $P_{\mathrm{use}}\left(t\right)$ is instantaneous energy expenditure (W), Δt is the simulation time step (s), and $E_{\text{in}}\left(t\right)$ is energy intake within the step (J). In this reserve-based operationalization, the conceptual notion of viability is measured by whether the system remains within the admissible energy constraint $E\left(t\right)>0$. Episodes terminate when $E\left(t\right)\leq 0$ or when a maximum horizon $T_{\max}$ is reached; survival time $T_{\text{surv}}$ is the elapsed time until termination. Energy and viability are therefore not equivalent concepts: energy is the accounted physical quantity, while viability is the persistence condition assessed through that accounting.

In Eq. (1), $E_{\text{in}}\left(t\right)$ should be understood as net energy intake unless otherwise specified. In biological foraging or untethered robotic operation, energy acquisition is itself an active process that can require search, movement, docking, processing, conversion, or storage. The intake term can therefore be decomposed as

$$E_{\text{in}}\left(t\right)=E_{\text{gain}}\left(t\right)-E_{\mathrm{acq}}\left(t\right)$$ (2)

where $E_{\text{gain}}\left(t\right)$ is the gross energy obtained from food, charging, solar harvesting, or other environmental sources, and $E_{\mathrm{acq}}\left(t\right)$ is the energetic cost of acquiring that energy. This distinction matters because positive gross intake does not guarantee viability if acquisition costs exceed the energy gained.

Thus, $E\left(t\right)>0$ is not a second definition of viability, but the experimental criterion used to measure persistence under the specified accounting rule. This operationalization is compatible with viability theory, which studies whether system trajectories remain within admissible constraints over time [64].

Eq. (1) is intentionally general. Its scientific value is that heterogeneous contributions must pass through the same accounting constraint, making trade-offs interpretable rather than implicit. This is the core sense in which energy functions as a common currency for embodied evolution.

**Fig. 3.**
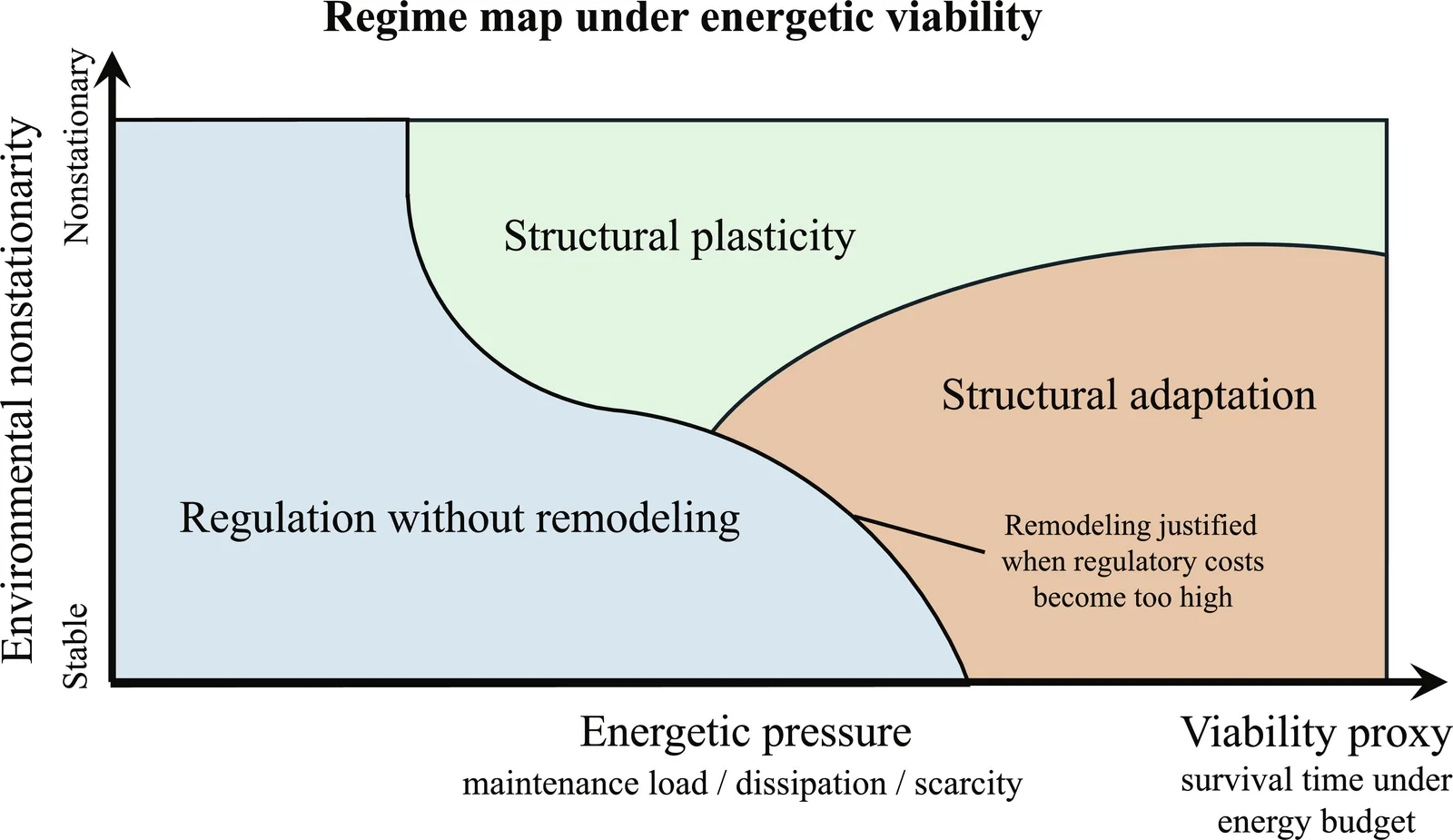
Biology-facing regime schematic under energetic viability. Energetic constraint can organize 3 broad adaptive responses: regulation without remodeling, structural adaptation, and structural plasticity. Remodeling or plasticity may become advantageous when regulatory costs become too high or when environmental nonstationarity makes fixed responses insufficient.

Fig. 3 summarizes a central hypothesis enabled by explicit accounting: different adaptive responses can be energetically justified under different energetic and environmental conditions. The framework does not assume any mechanism is universally superior. Instead, it motivates identifying when regulation without remodeling is sufficient, when structural adaptation becomes worthwhile, and when structural plasticity with explicit energetic cost is justified under nonstationary energetic structure. The regimes differ in which components may change, when those changes occur, and which energetic costs must be represented, consistent with timescale analyses of embodied intelligence [65].

The boundaries in Fig. 3 are schematic rather than fitted phase boundaries. They indicate hypothesized transitions in the dominant adaptive mechanism as energetic pressure and environmental nonstationarity increase. When energetic pressure is low and the environment is stable, regulation without remodeling may be sufficient because compensatory control remains affordable. As energetic pressure increases, structural adaptation can become advantageous when a changed body–controller organization reduces long-run expenditure. When environmental nonstationarity is high, structural plasticity becomes relevant because fixed morphology or across-generation adaptation may be too slow to maintain viability. Thus, the map is partitioned according to which class of mechanism is expected to provide the lowest-cost route to persistence under the specified accounting rule.

A viability framework only functions as a common currency if it accounts for the energetic costs of acting and persisting as a body. In biology, persistent maintenance costs and allocation trade-offs are foundational [15,40,41]. In embodied robots and simulations, the analogous requirement is that accounting includes at least an action-dependent expenditure term (actuation and control effort), a baseline overhead term (constant losses), and a maintenance-like term that prevents morphological complexity from being implicitly free over long horizons. When morphology can change within the lifetime (remodeling, repair, and reconfiguration), an additional principle becomes relevant: the energetic cost of adaptation should be included explicitly as an energetic commitment; otherwise, online morphological change becomes an unrealistically free operation. This is biology-consistent because repair and regeneration are energetically costly and induce allocation trade-offs [35–37].

To instantiate the framework concretely while remaining general, we use a reference expenditure model that combines actuation-related expenditure with baseline overhead and a maintenance-like term:

$$\begin{array}{c} P_{\mathrm{use}}\left(t\right)\;=\\ \frac{1}{\eta }\sum_{i}\left|\tau_{i}\left(t\right)\;{\dot{q}}_{i}\left(t\right)\right|\;+\;k_{\mathrm{cu}}\sum_{i}\tau_{i}^{2}\left(t\right)\;+\;P_{\text{idle}}\;+\;\lambda_{b}m^{\alpha } \end{array}$$ (3)

In Eq. (3), $\tau_{i}\left(t\right)$ is the torque at joint i (N·m), and ${\dot{q}}_{i}\left(t\right)$ is the time derivative of joint angle $q_{i}$ (rad/s). The product $|\tau_{i}\left(t\right){\dot{q}}_{i}\left(t\right)|$ is therefore the magnitude of instantaneous mechanical power at joint i (W), and the summation gives the action-dependent mechanical power across all actuated joints. The factor $\eta \in \left(0,\;1\right]$ is an effective efficiency that maps mechanical power demand to modeled energetic expenditure.

The second term, $k_{\mathrm{cu}}\sum_{i}\tau_{i}^{2}\left(t\right)$, is an actuation-effort regularizer that captures energetic burden not represented by mechanical work alone; $k_{\mathrm{cu}}$ has units chosen so that this term yields power (W). $P_{\text{idle}}$ is a constant baseline overhead (W), representing cost incurred even when the agent is not actively moving. The final term, $\lambda_{b}m^{\alpha }$, is a maintenance-like cost (W) that increases with a morphology-dependent proxy m, such as total mass, material volume, or an equivalent structural measure. The exponent α specifies the scaling relation, and $\lambda_{b}$ has units chosen so that $\lambda_{b}m^{\alpha }$ yields power (W).

In practice, η, $k_{\mathrm{cu}}$, $P_{\text{idle}}$, $\lambda_{b}$, and α are modeling parameters that can be set from measurements when available or chosen as controlled experimental factors in simulation studies. In fixed-morphology regimes, m is constant within an episode; in morphology-changing regimes, m varies with the embodiment and may vary within an episode when remodeling is enabled. Together, these terms ensure that $P_{\mathrm{use}}\left(t\right)$ is expressed in watts and is consistent with the reserve update in Eq. (1).

The intent is not to claim biological realism of a single scaling exponent; metabolic scaling remains debated and context-dependent [31,47–51]. Rather, the maintenance-like term encodes the biologically grounded principle that persistence has a cost and that larger or more materially intensive embodiments carry larger baseline energetic liabilities ([15], Chapter 2, and [16], Chapters 15 to 25) [63].

For cross-embodiment comparison, energetic accounting must be transparent. At minimum, studies should state the reserve update and termination criterion, how expenditure is decomposed (action, overhead, maintenance, and adaptation if applicable), and how intake and dissipation are defined in the environment. These commitments are lightweight but essential if persistence-based conclusions are to be interpreted and compared across embodiments and platforms.

## Illustrative Experimental Paradigm and Evidence

**Fig. 4.**
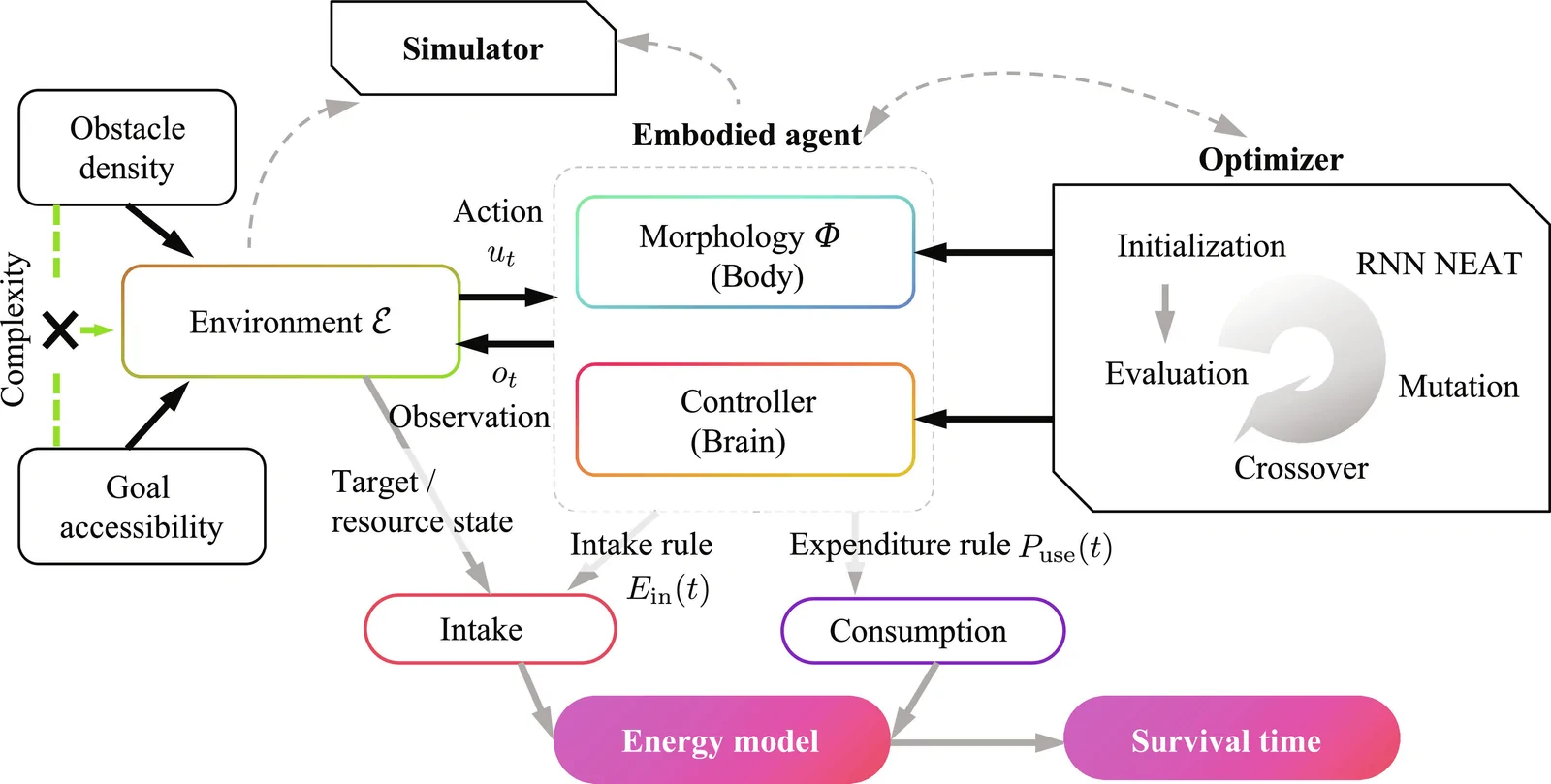
Illustrative pipeline for energy-centered evaluation. The agent acts on the environment, actions generate expenditure, and target/resource events generate intake through an explicit intake rule. The accounting module updates the internal reserve using Eq. (1), and survival time measures persistence under energetic constraint.

**Fig. 5.**
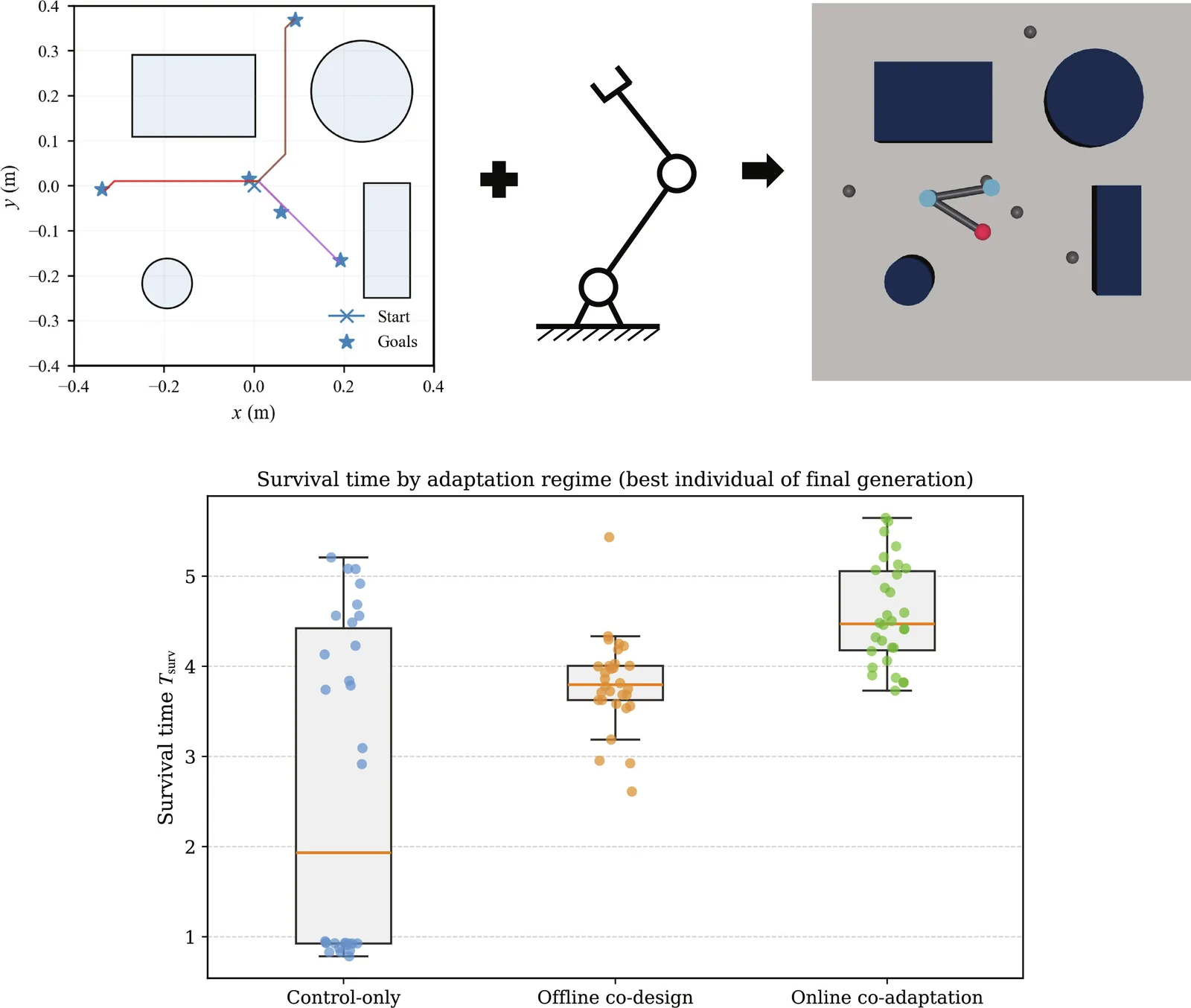
Illustrative evidence for energy-centered evaluation under a shared reference energy model. The top panel shows the planar-arm reaching setup: a bounded workspace with obstacles, start position, target sequence, articulated arm, and corresponding simulation environment. Target acquisition triggers energy intake according to $E_{\text{in}}\left(t\right)=\Delta E_{\text{target}}$, while movement and morphology-dependent persistence costs reduce the reserve. The bottom panel shows survival time $T_{\text{surv}}$ for the best individual of the final generation across 3 adaptive regimes: control-only, offline co-design, and online co-adaptation. All regimes are evaluated under the same task, target sequence, intake rule, initial energy budget, and expenditure model, so differences indicate persistence under shared energetic constraint rather than differences in task definition.

Figs. 4 and 5 summarize the energy-centered evaluation pipeline and report the illustrative comparison, respectively.The top panel shows the planar-arm reaching setup, including the workspace, obstacles, start position, target sequence, articulated arm, and corresponding simulation environment. The agent receives energy only when the end-effector reaches a target within the tolerance radius, while movement and morphology-dependent terms deplete the reserve. The bottom panel reports survival time for the best individual of the final generation under 3 adaptive regimes: control-only, offline co-design, and online co-adaptation. The comparison should be interpreted as configuration-dependent evidence for the value of energy accounting, not as a universal ranking of regimes.

The relative ranking shown in the bottom panel should be interpreted as configuration-dependent. Changes in morphology, controller capacity, target distribution, intake magnitude, initial reserve, maintenance coefficient, or environmental difficulty may alter the observed survival-time pattern. Therefore, the figure is intended as an illustrative comparison under one shared accounting configuration rather than a universal ranking of adaptive regimes. More extensive benchmark studies should evaluate sensitivity across multiple configurations and report uncertainty over independent random seeds.

The previous sections argued that energy can serve as a commensurate currency for embodied evolution and motivates hypothesis-level regime structure (Fig. 3). This section first presents a planar-arm illustration of how the proposed currency can be instantiated and reported in practice, and then summarizes a supplementary cross-platform check using an additional fixed-morphology mobile robot. The purpose is not to present a definitive benchmark result, but to show how a robotic simulation can function as a controlled biological model system: the task, body, controller, environment, intake rule, and expenditure model are specified so that persistence under energetic constraint can be compared across adaptive regimes. In this sense, explicit energy accounting makes embodied costs visible and supports interpretable comparisons between regimes under a shared accounting principle [12,20].

The illustrative task is sequential target reaching with a planar robotic arm. In each episode, the arm must reach a series of target locations within a bounded workspace (top panel of Fig. 5). We compare 3 adaptive regimes under the same environment and energy accounting: (i) regulation without remodeling (control-only), where arm morphology is fixed and only the controller is optimized; (ii) structural adaptation (offline co-design), where morphology and controller are optimized across design iterations or generations but remain fixed within an episode; and (iii) structural plasticity (online co-adaptation), where limited within-episode remodeling is permitted (subject to the same accounting of expenditure and maintenance) and evaluated under the same energy accounting. Within each evaluated episode, the morphology and controller are evaluated under the same environment, target sequence, intake rule, and energy-accounting model, so the reported differences reflect persistence under a shared energetic constraint rather than changes in task definition.

Energy intake is defined by an explicit rule tied to the task. When the end-effector reaches a target within a tolerance radius, the agent receives an energy increment $E_{\text{in}}\left(t\right)=\Delta E_{\text{target}}$; otherwise, $E_{\text{in}}\left(t\right)=0$. The same intake rule is used for all regimes. This simplified intake rule is a net-intake abstraction. It captures the energetic consequence of target acquisition but does not explicitly model the additional search, docking, harvesting, conversion, or storage costs that a biological organism or untethered robot would pay to obtain energy. In applications where energy acquisition is itself a behavior, $E_{\text{in}}\left(t\right)$ should be decomposed into gross gain and acquisition cost as in Eq. (2).

Fig. 4 summarizes the experimental loop and clarifies how energy is obtained from the environment. During an episode, the agent sends control actions to the environment; these actions produce movement and action-dependent expenditure. The environment returns task feedback, including whether the end-effector has reached a target or resource state. Energy intake is therefore generated through an explicit intake rule rather than assumed as an automatic input: in the reaching task, target acquisition within the tolerance radius sets $E_{\text{in}}\left(t\right)=\Delta E_{\text{target}}$, whereas nonacquisition sets $E_{\text{in}}\left(t\right)=0$. This intake is combined with expenditure in Eq. (1) to update the internal reserve. Thus, the terms previously indicated schematically as “where” and “how” correspond respectively to the target/resource state in the environment and the intake rule that maps environmental events to energetic gain. Episodes start with $E\left(0\right)=E_{0}$ and terminate when $E\left(t\right)\leq 0$ or when $T_{\max}$ is reached; survival time $T_{\text{surv}}$ is the elapsed time until termination.

The map-sharing component is included to ensure that episode outcomes are not interpreted as isolated scores. Each run is placed onto the same regime map introduced in Fig. 3, which distinguishes regulation, structural adaptation, and structural plasticity. Formally, map sharing means that all regimes are evaluated under a common state–accounting–interpretation structure: the same environmental task defines interaction states, the same energy model defines reserve dynamics, and the same regime map defines how outcomes are categorized for comparison.

To support replication, we report the environment layout and target sequence; controller architecture and optimization budget for control-only; morphology parameterization and mutation/remodeling rules for the other regimes; energy parameters $\left(E_{0},\;P_{\text{idle}},\;\lambda_{b},\;\alpha ,\;\eta ,\;k_{\mathrm{cu}},\;\Delta E_{\text{target}}\right)$; time step Δt; termination criteria $\left(E\leq 0\;\text{or}\;T_{\max}\right)$; number of trials N and random seeds; and the survival-time summary statistic (mean ± SD across trials).

Expenditure is computed using a shared reference accounting model [Eq. (3)] that combines action-dependent cost with baseline overhead and a maintenance-like term that accounts for persistence. The inclusion of explicit maintenance is biologically motivated, while acknowledging that detailed scaling forms are debated and context-dependent [31,47–51]. The key point is methodological: a shared accounting rule makes it possible to compare embodiments and regimes without collapsing them into task-specific reward semantics.

Task performance can correlate with energy use in some settings, but it does not generally subsume explicit accounting because it typically omits baseline overhead, morphology-dependent persistence costs, and the duration over which task achievement can be sustained. Under shared accounting and environment conditions, we therefore report both targets reached and survival time (Table 2). This comparison is not intended to prove that energy accounting is universally better than task scoring; rather, it shows that task achievement and energetic persistence are complementary outcomes that should be reported together when the target construct is sustained embodied organization.

**Table 2.** Task achievement versus energetic persistence under shared accounting

Regime	Targets reached (mean ± SD)	$T_{\text{surv}}$ (mean ± SD)
Control-only	1.80 num±1.88 num	2.59 s±1.79 s
Offline co-design	3.63 num±0.72 num	3.81 s±0.51 s
Online co-adaptation	4.00 num±0.00 num	4.57 s±0.57 s

Table 2 illustrates how task achievement and energetic persistence can be compared without treating one as a substitute for the other. In this example, online co-adaptation reaches the largest number of targets and also achieves the longest survival time, while the relative changes across regimes are not identical for the 2 measures. For instance, moving from control-only to offline co-design produces a larger proportional improvement in targets reached than in survival time, indicating that task achievement and persistence do not necessarily scale in the same way. The comparison therefore supports the main methodological point: explicit energy accounting adds information about how long an embodied organization can remain viable under cost, even when task metrics are also reported.

This comparison demonstrates the specific limitation of task-only evaluation targeted by the framework. Task achievement identifies how many goals are completed, whereas survival time identifies how long the embodied organization remains viable under the same intake, expenditure, and reserve dynamics. The 2 measures therefore answer different questions: targets reached describe episodic success, while survival time under explicit accounting describes persistence under embodied cost.

For readability, we label the 2 environment configurations as Env-L and Env-H in the main text. Env-L denotes the lower-pressure environment, whereas Env-H denotes the higher-pressure environment. Rover is a fixed-morphology 4-wheel mobile rover with 2 actuated front-wheel joints and 2 passive rear-wheel joints; it is used here as a mobile-navigation platform to test whether the energy-accounting framework remains interpretable when the embodiment differs from the articulated Arm. Detailed platform definitions, environment parameters, internal identifiers, and reporting protocols are provided in the Supplementary Materials.

Table 3 complements the aggregate Arm comparison in Table 2 by reporting a targeted 2-platform, 2-environment validation. The Arm and Rover differ structurally and operationally: the Arm emphasizes articulated reaching and joint-level actuation costs, whereas Rover emphasizes mobile navigation, path length, and environment-dependent traversal costs. This design separates 3 comparisons: Arm strategies are compared within identical environment and accounting settings; each platform is evaluated across Env-L and Env-H to test environment sensitivity; and Rover provides an embodiment change under the shared reporting protocol. The results show that task achievement and energetic persistence remain distinct outcomes across platform and environment changes. For Rover, moving from Env-L to Env-H reduces both targets reached and survival time under E2 accounting. For the Arm, all 3 strategies reach 4 targets in Env-L, yet survival time differs across strategies; in Env-H, online co-adaptation reaches the most targets and also achieves the longest survival time. These results do not establish a universal ranking of platforms or adaptive regimes. Instead, they show that explicit accounting can report task achievement and energetic persistence across different embodied systems without collapsing them into a single task score.

**Table 3.** Cross-platform 2-environment validation and Rover energy-model comparison. Values are mean ± sample SD across 5 random seeds. Env-L and Env-H denote the lower-pressure and higher-pressure environment configurations, respectively.

Platform	Environment	Strategy	Model	Targets reached	$T_{\text{surv}}$/s	Coverage entropy
Arm	Env-L	Control-only	E2	4.00±0.00	4.61±0.31	2.34±0.44
Arm	Env-L	Offline co-design	E2	4.00±0.00	3.98±0.03	1.76±0.02
Arm	Env-L	Online co-adaptation	E2	4.00±0.00	4.11±0.23	2.94±0.27
Arm	Env-H	Control-only	E2	2.80±0.45	3.48±0.44	1.96±0.35
Arm	Env-H	Offline co-design	E2	2.40±0.89	3.15±0.47	1.43±0.88
Arm	Env-H	Online co-adaptation	E2	4.00±0.00	4.86±0.60	1.99±0.40
Rover	Env-L	Control-only	E1	0.20±0.45	20.00±0.00	1.07±0.87
Rover	Env-L	Control-only	E2	3.20±0.84	3.39±0.59	2.79±0.49
Rover	Env-H	Control-only	E1	0.20±0.45	20.00±0.00	0.82±0.35
Rover	Env-H	Control-only	E2	2.00±0.00	2.53±0.05	1.54±0.30

The E1 rows for Rover are included to show the sensitivity of interpretation to the accounting model. Because E1 does not include the basal-metabolism or maintenance-like term used in E2, Rover survival under E1 reaches the 20-s episode cap and is therefore right-censored by the evaluation horizon. We therefore use the E2 rows as the main cross-platform energetic-viability comparison, while treating E1 as an accounting-model sensitivity check. Coverage entropy is reported as an exploration descriptor; because the Arm and Rover use different tracked points, coverage grids, bounds, and footprint handling, this quantity supports within-platform interpretation rather than strict absolute platform ranking.

Together, the planar-arm illustration and the cross-platform 2-environment validation highlight why energy accounting can strengthen robots and simulations as biological models. First, it makes embodied cost visible: strategies that look similar under task-only scoring can diverge once persistence, action costs, and maintenance-like costs are explicitly accounted for, because aggressive compensation and energetically expensive structures may shorten survival under a fixed budget [53,54,56,66,67] ([57], Chapters 1 to 3, 7, and 19).

Second, it supports mechanism-level comparison: structural adaptation can increase survival by shifting burden from continual regulation into passive structure and improved mechanical efficiency [3,17,60,68]. Third, it identifies what future evaluations must make explicit: if structural plasticity, remodeling, repair, and growth are enabled, their energetic costs should be represented rather than treated as free operations, consistent with regeneration as a costly, trade-off-laden process in biology [35–37].

## What Changes When Energy Is the Currency

The framework above changes what embodied evolution can compare and therefore what it can explain. Under task-centered evaluation, results are anchored to task semantics and can be difficult to generalize across embodiments or environments. Under energy-accounted evaluation, outcomes are interpretable as persistence under constraint, and contributions from morphology, behavior, and environment become comparable through a shared physical currency [12,20]. Three consequences are as follows.

First, sustainability becomes the primary interpretation frame. A strategy that achieves a goal with high expenditure can be ranked superior under task-centered fitness even if it would fail over longer horizons or under small perturbations. Under persistence under energetic constraint, task achievement remains meaningful but is subordinate to whether the underlying organization can be sustained under ongoing energetic pressure. This aligns with biological perspectives in which persistence requires continual payment of maintenance and regulatory costs [15,33,34,40] and with biomechanics evidence that sustained function is organized by measurable energetic costs [53,54,56,66]. In practice, energy-centered evaluation makes it possible to distinguish strategies that are effective but energetically brittle from strategies that are viable and sustainable, even when both achieve similar task outcomes [69–71].

Second, complexity becomes interpretable rather than implicit. Task performance alone provides weak guidance for understanding whether additional degrees of freedom, actuation, or controller sophistication are beneficial in a principled sense. Energetic viability makes the trade-off explicit: larger bodies, more active actuation, and continual corrective control incur ongoing cost that directly affects survival [30,31]. This does not imply that simpler is always better; it supports a distinction between effective complexity, which reduces long-run expenditure through passive dynamics or mechanical advantage, and gratuitous complexity, meaning added degrees of freedom or activity that improve task score while increasing persistent energetic burden without improving persistence. Biomechanics and neuro-mechanics provide concrete mechanisms for such differences, for example, when structural organization reduces collision losses and hence metabolic cost [71,72], and robotics provides canonical demonstrations in passive-dynamic and morphology-aware designs [3,17,60]. A viability lens also complements repertoire-based approaches: mapping diverse viable organizations can reveal how morphology, regulation, and complexity co-vary under energetic constraint rather than selecting a single winner [73–75].

Third, performance mechanisms become condition-dependent rather than universally ranked. Energetic viability suggests a more informative view: regulation without remodeling, structural adaptation, and structural plasticity are alternative mechanisms whose value depends on energetic pressure and environmental nonstationarity (Fig. 3). In low energetic pressure and stable environments, regulation without remodeling may be sufficient because compensatory regulation remains affordable [33]. As energetic pressure increases, structural adaptation can become justified because changes in morphology, transmission, and passive dynamics can improve mechanical efficiency and reduce long-run expenditure ([16], Chapters 23 to 25) [60]. Under high nonstationarity, structural plasticity can become valuable, but only when remodeling is treated as an energetic commitment; otherwise, online structural change becomes a free and biologically unrealistic operation. Biology provides strong precedent that repair and regeneration can sustain function but impose real energetic costs and trade-offs [35–37].

Taken together, these consequences suggest a shift in what should be reported as results in embodied evolution studies. Alongside task performance, studies should report viability statistics, energy decomposition (action, overhead, and maintenance), and the energetic structure of the environment (intake rules and dissipation). Without such reporting, conclusions about morphology, control, and adaptive mechanisms remain difficult to compare across embodiments and platforms [22,23,76,77].

## Open Challenges and Future Directions

This review argues that explicit energy accounting provides a physically grounded basis for comparing how regulation, morphology, and environment jointly produce adaptive organization. The remaining question is what must be resolved for energy-centered evaluation to become a mature, biology-facing research program in which robots and simulations function as controlled model systems rather than task solvers.

A first challenge is methodological discipline: energy-based conclusions are only comparable when accounting assumptions are explicit. At minimum, studies should state the accounting equations and termination criteria; the decomposition of expenditure into action, overhead, maintenance, and (in future work) remodeling; and the intake and dissipation structure imposed by the environment.

A second challenge is to treat the environment as the source of energetic structure. The regime schematic in Fig. 3 becomes scientifically meaningful only if energetic pressure and nonstationarity are manipulated deliberately rather than treated as incidental background. This shifts the experimental question from which agent performs best on a fixed task to when the dominant adaptive response changes as dissipation, scarcity, and resource accessibility are varied. Here, robotics has distinctive value for biology: constraints can be perturbed and controlled in ways that are difficult to isolate in living systems while preserving the physical couplings that make energetic trade-offs meaningful.

A third challenge is mechanistic interpretation. The energy-currency framing predicts that persistence is not improved only through better regulation. It can also arise through structural efficiency, passive dynamics, and body–environment coupling that reduce the energetic burden of continual compensation. A productive direction is therefore comparative: under shared accounting, when does a structure-first organization yield higher persistence than a regulation-first organization, and when does the advantage reverse under perturbation. Advancing beyond endpoint metrics will require linking expenditure components to specific morphological and policy features so that observed differences support mechanistic explanation rather than post hoc ranking.

A fourth challenge is to model energy acquisition as an active process rather than as an automatic reward. In biological systems, foraging consumes time and energy; in untethered robotic systems, recharging, docking, solar harvesting, or environmental energy collection also imposes costs and constraints. Future energy-centered evaluations should therefore distinguish gross intake from net intake by accounting for the energetic and temporal cost of searching for, accessing, converting, and storing energy. This extension is especially important for battery-powered and energy-harvesting robots operating without constant external supply, where autonomy depends not only on reducing expenditure but also on whether the agent can obtain energy at a net positive return. In such settings, energetic viability should be evaluated through closed-loop energy acquisition: the robot must spend energy to reach or harvest energy, and persistence depends on the balance between acquisition cost, conversion efficiency, storage capacity, and environmental resource distribution.

A final challenge is biological realism about plasticity. If within-lifetime structural change is allowed in robot model systems, its energetic cost must be represented; otherwise, plasticity becomes a free modeling degree of freedom and the regime logic loses meaning. This motivates a concrete empirical target: the remodeling threshold. The field should quantify when continued compensation becomes energetically dominated by structural change, how that threshold shifts with environmental structure, and whether explicit remodeling cost yields the predicted transition between regulation, structural adaptation, and structural plasticity.

These directions clarify what is at stake. Energy as a common currency is not simply a different metric; it is a way to make sustainability, trade-offs, and mechanism selection empirically visible in embodied evolution. If explicit accounting and environment-structured experiments become standard, robots and simulations can support biology-facing questions about how regulation, structure, and plasticity are selected under constraint, and answer them through controlled, physically grounded interventions.

## Data Availability

The data and materials used to support the findings of this study are available from the corresponding authors upon reasonable request.
