# Supplementary material for: Energy as a Common Currency for Understanding Embodied Evolution
Source: Cyborg Bionic Syst. 2026 Sep 16;7:0680. doi: 10.34133/cbsystems.0680 (PMC13579111; doi:10.34133/cbsystems.0680)
Supplement: Supplementary file 1 [file cbsystems.0680.f1.pdf]

# Supplementary Experimental Description

The two-platform comparison was designed to change embodiment while retaining a similar control dimensionality and mass scale. The Arm is a fixed-base planar 2R manipulator with two actuated revolute joints and two torque outputs. Rover is a mobile four-wheel rover with two actuated front-wheel joints and two passive rear-wheel joints, and its controller likewise produces two torque outputs. Rover has a total modeled mass of approximately 0.75 kg, while the Arm mass is obtained from its MuJoCo model and varies with link design. Both are therefore sub-kilogram systems of comparable mass order, rather than platforms separated by an order-of-magnitude mass difference. They use the same workspace, obstacles, energy-source coordinates, energy reserve, and optimization protocol. The comparison nevertheless does not make their kinematic tasks identical: the Arm routes an end effector around obstacles, whereas Rover must translate its full chassis and wheel footprint. Consequently, the results primarily support comparisons within each platform and a cross-platform demonstration of common energy accounting, rather than a strict ranking of the two robots.

Table S1: Comparison of the two embodied platforms.

| Property | Arm | Rover |
|---|---|---|
| Embodiment | Fixed-base planar 2R manipulator with two links | Four-wheel mobile rover with a rigid chassis |
| Actuated joints | 2 revolute joints | 2 driven front-wheel joints |
| Additional passive joints | None | 2 passive rear-wheel joints |
| Planar mobility | End-effector motion generated by two joint angles | Chassis $x$, $y$, and yaw generated by differential front-wheel torque |
| Modeled mass | MuJoCo-derived and morphology-dependent | 0.75 kg total |
| Energy collector | End effector | Chassis centre |
| Strategies | Control-only, offline co-design, and online co-adaptation under E2 | Fixed-morphology control-only under E1 and E2 |

Table S2: Experimental-factor comparison.

| Factor | Level | Definition | Role in the comparison |
|---|---|---|---|
| Energy model | E1 | $P_{\text{use}}^{\text{E1}} = \sum_i \lvert\tau_i\omega_i\rvert/\eta + k_{\text{cu}} \sum_i \tau_i^2 + P_0 + k_w \sum_i \omega_i^2$ | Motion-related accounting without stationary basal drain |
| Energy model | E2 | $P_{\text{use}}^{\text{E2}} = \sum_i \lvert\tau_i\omega_i\rvert/\eta + k_{\text{cu}} \sum_i \tau_i^2 + P_{\text{idle}} + \lambda_b m^{\alpha}$ | Adds morphology-dependent stationary maintenance |
| Environment | Env-L | obstacle density 0.05, detour ratio 1.0, and one cylinder centred at $(-0.163, -0.079)$ m, with radius approximately 0.0546 m. | Sparse, lower-pressure environment. |
| Environment | Env-H | obstacle density 0.20, detour ratio 1.0, three cylinders with radii 0.0431–0.0465 m, and two boxes with planar footprints about $0.050 \times 0.109$ m and $0.130 \times 0.051$ m. | More cluttered, higher-pressure environment. |

The five energy-source coordinates are identical for the two robots within each environment. In Env-L they are $(0.199, -0.131)$, $(-0.016, -0.107)$, $(0.155, 0.132)$, $(-0.095, 0.123)$, and $(0.090, 0.054)$ m. In Env-H they are $(0.239, 0.068)$, $(0.134, -0.036)$, $(-0.041, -0.221)$, $(-0.125, -0.259)$, and $(0.046, -0.141)$ m. Obstacles are solid collision geometries while energy sources are non-colliding markers that supply energy only on their first hit.

Table S3: Shared simulation, optimization, and reporting protocol.

| Item | Setting |
|---|---|
| Time step | $\Delta t = 0.001\,\text{s}$ |
| Start pose | $(0, 0, 0)$ |
| Energy reserve | $E_0 = 3$, bounded to $[0, 30]$ |
| Energy sources / intake | Five sources; first hit on each source adds +4 |
| Hit radius | Arm 0.020 m; Rover 0.025 m |
| Episode termination | Energy reaches zero, or $T_{\max} = 20\,\text{s}$ (20,000 steps) |
| Controller / optimization | Recurrent NEAT; 300 generations; population 20 |
| Independent runs | Seeds 0–4 for each reported cell ($N = 5$) |
| Primary outcomes | Targets reached, survival time, and coverage entropy |
| Interpretation of coverage | Within-platform only because tracked points, grids, bounds, and footprint handling differ between Arm and Rover |

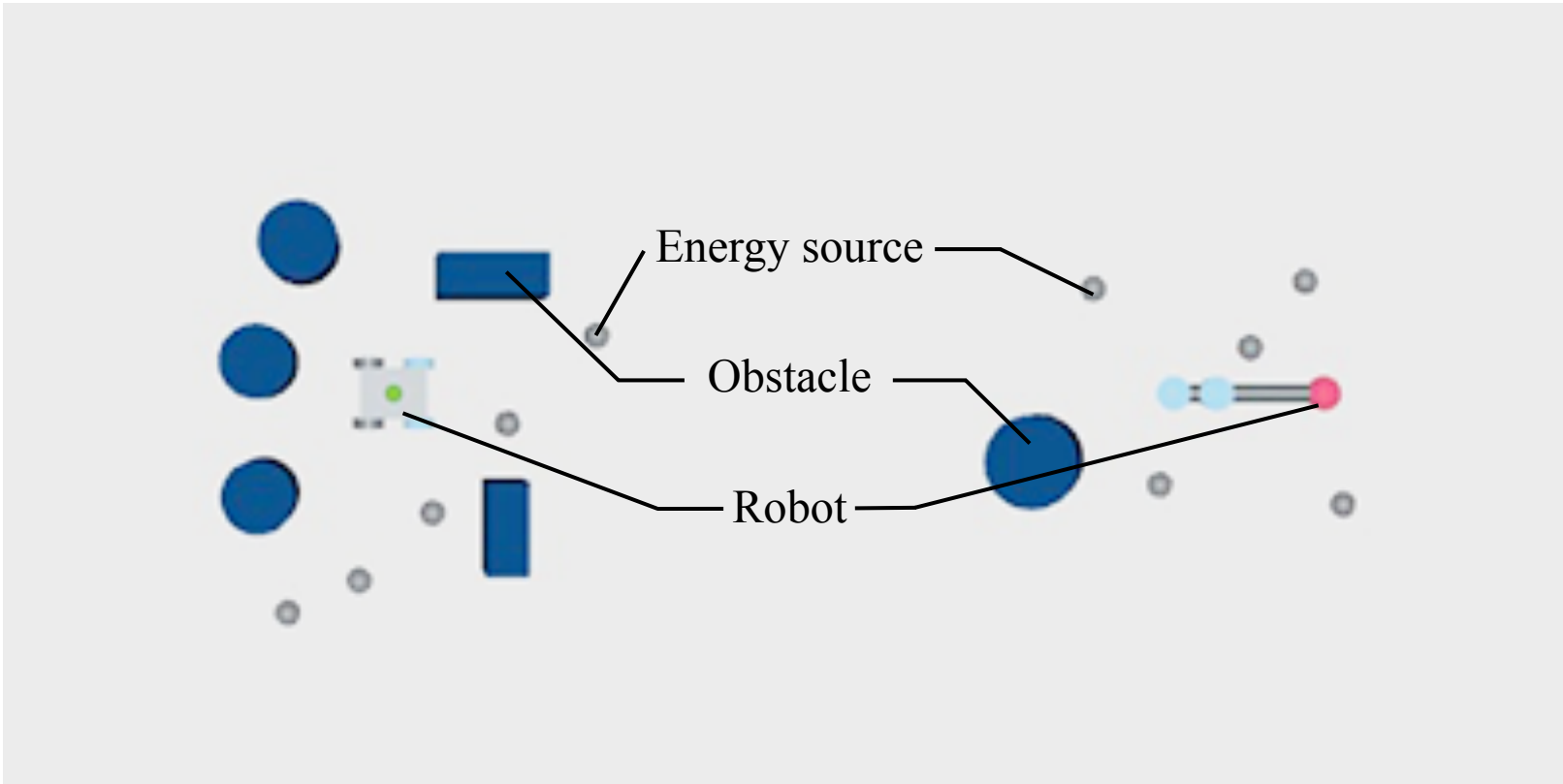


Figure S1: Shared environment representation for Rover (left) and the planar Arm (right). Both platforms use the same obstacle and energy-source layouts. The robot embodiment changes, while target-hit events, energy replenishment, and logging follow the shared protocol.

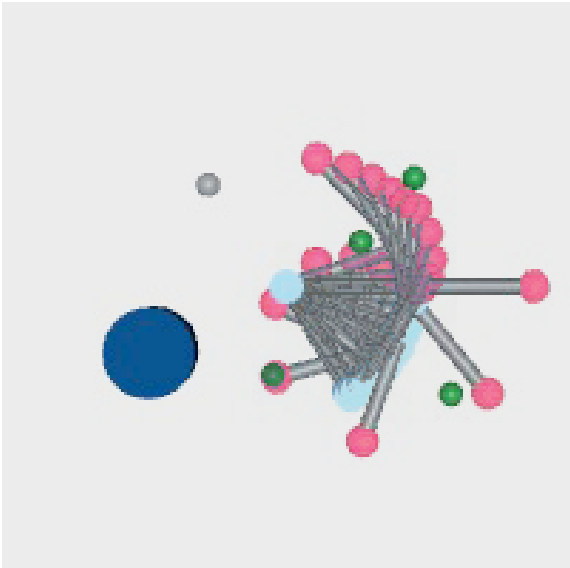

(a) Arm, E2 control-only.

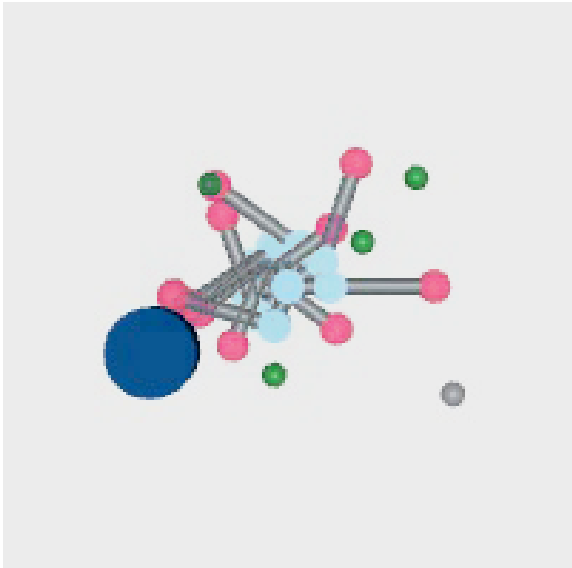

(b) Arm, E2 offline co-design.

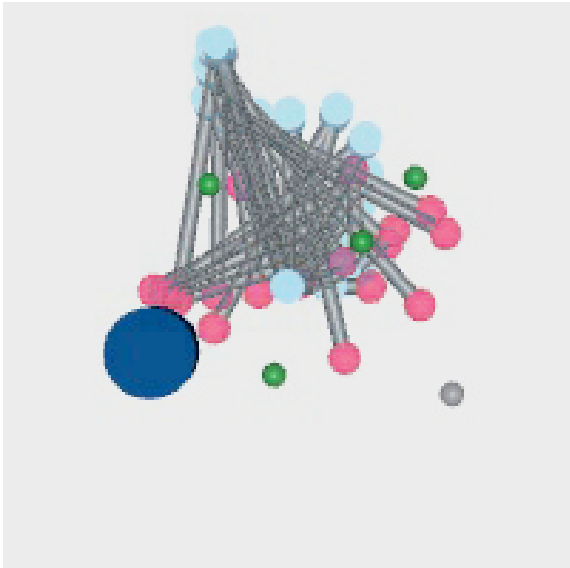

(c) Arm, E2 online co-adaptation.

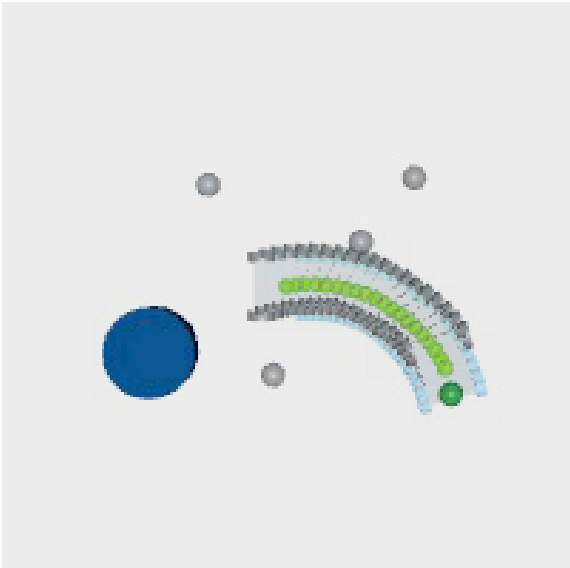

(d) Rover, E1 control-only.

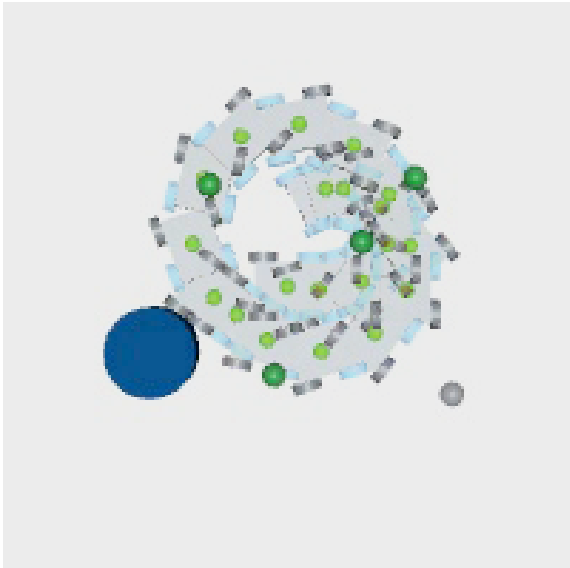

(e) Rover, E2 control-only.

Figure S2: Representative best-run motion plots in Env-L. Robot configurations show the swept motion over one representative run; the coloured markers indicate successively achieved energy source (dark green) or end-effector positions (red for Arm and light green for Rover).

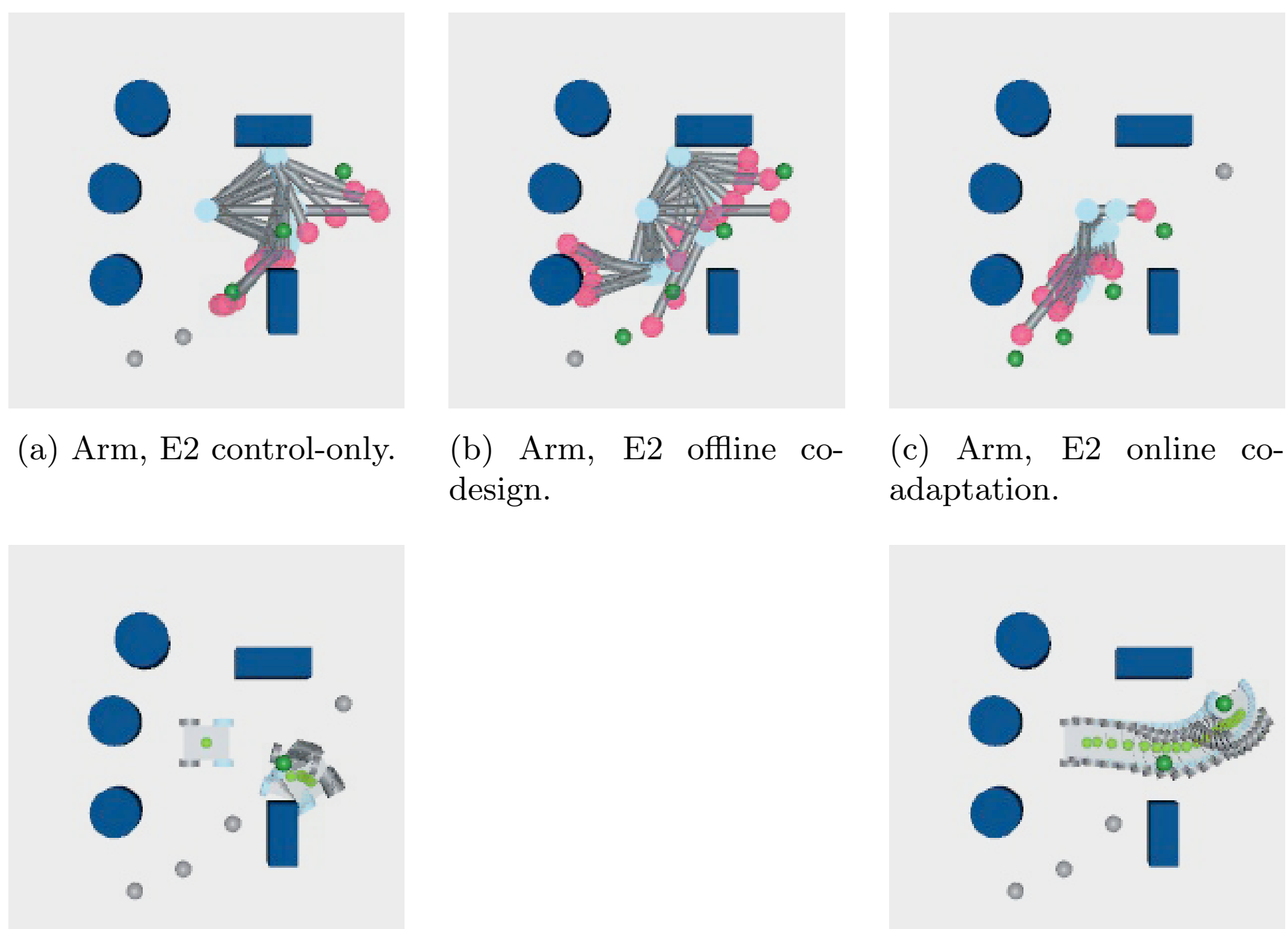

(a) Arm, E2 control-only.

(b) Arm, E2 offline co-design.

(c) Arm, E2 online co-adaptation.

(d) Rover, E1 control-only.

(e) Rover, E2 control-only.

Figure S3: Representative best-run motion plots in Env-H.
